# Newly emerging IADL impairment predicts subsequent cognitive decline across four population-based aging cohorts

**DOI:** 10.3389/fnagi.2026.1829741

**Published:** 2026-07-22

**Authors:** Xiaofan Xu, Jie Hao, Yan Fan, Yiming Huang, Zhenkun Huang, Huiyuan Zhang

**Affiliations:** 1 Department of Neurology, Liaocheng People’s Hospital, Liaocheng, Shandong, China; 2 Department of Neurology, Liaocheng Veterans Hospital of Shandong Province, Liaocheng, Shandong, China; 3 Department of Neurology, Xuanwu Hospital, Capital Medical University, Beijing, China

**Keywords:** cognitive decline, dementia risk, instrumental activities of daily living, longitudinal cohorts, neurocognitive aging

## Abstract

Scalable clinical markers of neurocognitive vulnerability in aging are needed, particularly in settings where biomarker access remains limited. Instrumental activities of daily living (IADLs) capture cognitively demanding everyday behavior and may provide a low-cost, repeatable functional marker of elevated risk. We harmonized longitudinal data from four population-based aging cohorts—CHARLS, ELSA, HRS, and SHARE—including adults aged ≥50 years with eligible baseline assessments. IADL impairment was defined as difficulty in at least one instrumental activity, and global cognition was harmonized within cohort as a standardized cognitive *z*-score. We first examined whether baseline IADL impairment was associated with subsequent cognitive slope, and then tested whether prior-wave IADL impairment predicted subsequent annualized cognitive change using lagged longitudinal models restricted to participants without baseline IADL impairment. In this context, newly emerging IADL impairment refers to IADL difficulty observed during follow-up among participants who were unimpaired at baseline. Dose–response analyses evaluated the number of impaired IADL domains. Cohort-specific estimates were generated separately and pooled using random-effects meta-analysis. Prior-wave IADL impairment predicted faster subsequent annualized cognitive decline (pooled *β* = −0.111 SD/year, 95% CI −0.141 to −0.081; *p* < 0.001). This corresponds to an expected difference of approximately −0.22 SD over 2 years, −0.44 SD over 4 years, and −0.67 SD over 6 years, assuming a constant linear difference in slope. A graded dose–response pattern was observed: compared with 0 impaired domains, 1 impaired domain was associated with *β* = −0.095 SD/year, ≥2 impaired domains with *β* = −0.147 SD/year, and each additional impaired domain with *β* = −0.063 SD/year. By contrast, baseline IADL impairment was not significantly associated with subsequent cognitive slope (*β* = 0.005 SD/year, 95% CI −0.002 to 0.011; *p* = 0.145). Findings were robust across sensitivity analyses and directionally consistent across cohorts, although effect magnitudes were heterogeneous. These findings support repeated IADL assessment as a pragmatic trigger for risk stratification and timely cognitive evaluation in initially functionally intact aging populations, rather than as definitive evidence of disease-specific neurodegenerative decline.

## Introduction

Dementia is a leading cause of disability and dependency in ageing societies, and the number of affected individuals is projected to rise markedly by 2050 (GBD 2019 Dementia Forecasting Collaborators, 2022). In the absence of curative therapies, increasing emphasis has been placed on prevention-oriented strategies that identify individuals at elevated risk as early as possible (Livingston et al., 2017, 2020, 2024). Although biomarker-based frameworks have substantially advanced the biological characterization of Alzheimer’s disease across its continuum (Sperling et al., 2011; Jack et al., 2016, 2018; McKhann et al., 2011; Dubois et al., 2014), access to biomarker testing remains uneven in routine clinical care and population settings (Karikari et al., 2020). Scalable, low-cost clinical signals that can be captured reliably in community and primary-care contexts are therefore needed to detect early neurocognitive vulnerability and support timely cognitive evaluation and prevention-oriented intervention (Livingston et al., 2017, 2020, 2024).

Instrumental activities of daily living (IADLs) reflect complex, cognitively mediated activities necessary for independent living, such as managing medications and finances, shopping, and meal preparation (Lawton and Brody, 1969). These tasks rely heavily on episodic memory, attention, and executive control, suggesting that subtle IADL difficulties may emerge during prodromal stages while basic self-care remains preserved. Accumulating evidence has challenged the traditional notion that mild cognitive impairment (MCI) is entirely functionally intact (Petersen et al., 1999; Winblad et al., 2004; Albert et al., 2011). A systematic review reported consistent IADL vulnerabilities in MCI (Jekel et al., 2015), and longitudinal studies have shown that functional deficits in MCI predict progression to dementia (Tabert et al., 2002; Luck et al., 2012). Community- and clinic-based studies further suggest that cognitively demanding IADL impairments can discriminate early cognitive vulnerability from normal ageing (Reppermund et al., 2013; Farias et al., 2006) and that specific Functional Activities Questionnaire items predict progression from clinically normal status to MCI (Pfeffer et al., 1982; Marshall et al., 2015). Biological plausibility also supports this link: IADL impairment has been associated with *in vivo* amyloid and tau burden (Halawa et al., 2019), and subtle functional difficulties relate to amyloid and cognition even among cognitively normal older adults (Marshall et al., 2020). Together, these findings support IADL assessment as a pragmatic early window into aging-related neurocognitive vulnerability and dementia risk (Huang et al., 2023).

Despite growing interest, several important questions remain unresolved. First, many studies assess IADL and cognition concurrently, limiting inference about whether functional impairment precedes subsequent cognitive deterioration. Second, longitudinal evidence is often derived from single cohorts or clinic-based samples, with limited cross-national replication. Cultural and contextual differences may influence both IADL reporting and its relationship with cognition, and cross-cultural adaptation of self-report measures is itself a non-trivial methodological issue (Beaton et al., 2000; Dubbelman et al., 2020b). Third, heterogeneity in IADL definitions and cognitive outcomes has limited comparability across studies. Finally, functional ability may change subtly over time in individuals with early cognitive vulnerability, highlighting the need for designs that explicitly model temporal ordering (Wadley et al., 2007).

To address these gaps, we leveraged harmonized measures across four large population-based ageing cohorts: CHARLS, ELSA, HRS, and SHARE (Zhao et al., 2014; Steptoe et al., 2013; Sonnega et al., 2014; Börsch-Supan et al., 2013). We hypothesized that IADL impairment at the prior wave would be associated with faster subsequent cognitive decline across cohorts, and that greater burden of IADL impairment would be associated with progressively faster decline. By applying a unified analytic framework across China, England, the United States, and continental Europe, this study aimed to clarify whether IADL difficulty emerging during follow-up among initially unimpaired participants can serve as a scalable functional marker for cognitive risk stratification in aging populations.

## Methods

### Study population and cohorts

We analyzed harmonized longitudinal data from four population-based ageing studies: the Health and Retirement Study (HRS) in the United States, the English Longitudinal Study of Ageing (ELSA) in England, the Survey of Health, Ageing and Retirement in Europe (SHARE), and the China Health and Retirement Longitudinal Study (CHARLS) (Zhao et al., 2014; Steptoe et al., 2013; Sonnega et al., 2014; Börsch-Supan et al., 2013). These cohorts are broadly comparable in design, with repeated assessments of health, cognition, and daily functioning conducted at approximately 2-year intervals. Their common structure enabled coordinated analyses across diverse populations of community-dwelling middle-aged and older adults.

**Figure 1:**
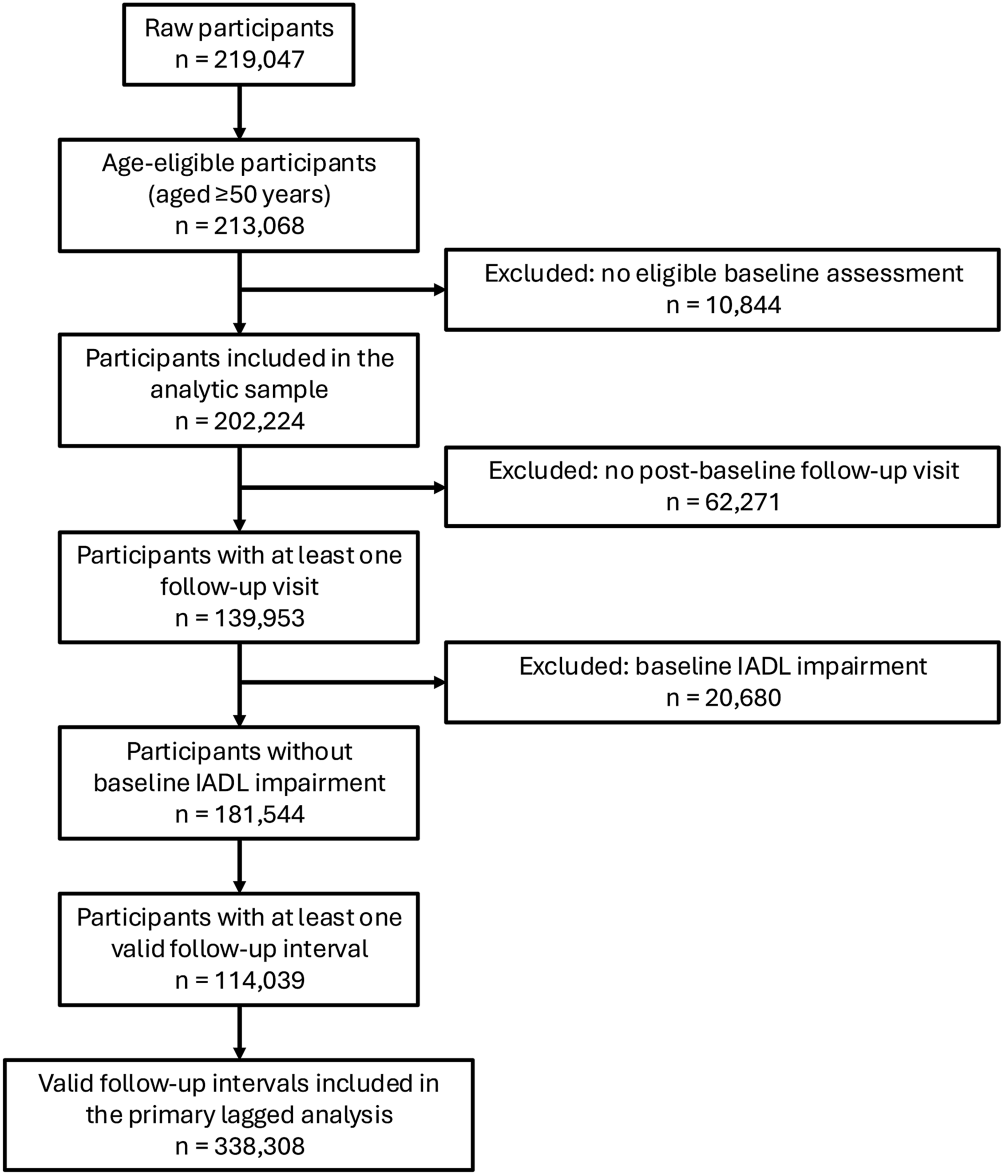
Flowchart of participant selection across the four population-based cohorts. Flow diagram showing the derivation of the analytic sample from CHARLS, ELSA, HRS, and SHARE. Participants were eligible if they were aged 50 years or older and had an eligible baseline assessment with usable data on instrumental activities of daily living (IADL), cognition, interview timing, and prespecified covariates. The figure summarizes exclusions and the final analytic sample used for longitudinal analyses.

Participants were eligible if they were aged 50 years or older and had an eligible baseline assessment. Eligible baseline was defined as the first wave with usable information on IADL status, cognition, interview timing, and prespecified covariates. Across the four cohorts, 202,224 participants met these criteria for the analytic sample. For the primary lagged analysis, participants additionally required at least one valid post-baseline follow-up interval; 114,039 individuals contributed 338,308 valid follow-up intervals. The derivation of the analytic sample is shown in Figure 1, with cohort-specific details provided in Supplementary Table S1. All original cohort studies received ethical approval from their respective institutional review boards, and all participants provided informed consent. The present study used de-identified public-use data.

## Cognitive and functional assessments

### Cognitive function

Each cohort administered repeated cognitive assessments at all study waves. Although the exact test batteries differed across cohorts, all cohorts included measures of immediate word recall, delayed word recall, and a brief mental-status/executive component. We harmonized cognition by constructing a cohort-specific global cognitive score (CogZ) at each wave. Within each cohort, component scores were standardized using the mean and standard deviation of the eligible baseline sample, and CogZ was calculated as the mean of available component *z*-scores when at least two of the three cognitive components were observed. Higher CogZ values indicate better cognitive performance. The descriptive score TR20 reported in Table 1 refers to total word recall, calculated as immediate recall plus delayed recall, and was not treated as a separate independent cognitive component in the CogZ construction. This distinction was made to avoid double-counting memory performance when constructing the global cognitive score.

**Table 1:** Baseline characteristics of participants included in the analytic sample across CHARLS, ELSA, HRS, and SHARE.

Characteristic	HRS	CHARLS	SHARE	ELSA
Total	33434	21072	130851	16867
Age, mean (SD)	61.65 (10.24)	59.64 (8.57)	64.22 (10.08)	61.56 (9.95)
50–64, *n* (%)	22469 (67.20)	15615 (74.10)	72856 (55.68)	11014 (65.30)
≥65, *n* (%)	10965 (32.80)	5457 (25.90)	57995 (44.32)	5853 (34.70)
Sex, *n* (%)				
Male	14471 (43.28)	10257 (48.68)	58775 (44.92)	7863 (46.62)
Female	18963 (56.72)	10815 (51.32)	72076 (55.08)	9004 (53.38)
Education, *n* (%)				
Below high school	7261 (21.72)	18276 (86.73)	53830 (41.14)	6171 (36.59)
High school	19190 (57.40)	2288 (10.86)	49531 (37.85)	7928 (47.00)
Above high school	6983 (20.89)	508 (2.41)	27490 (21.01)	2768 (16.41)
Smoking, *n* (%)				
No	27091 (81.03)	14772 (70.10)	105662 (80.75)	13816 (81.91)
Yes	6343 (18.97)	6300 (29.90)	25189 (19.25)	3051 (18.09)
Hypertension, *n* (%)				
No	18133 (54.24)	14992 (71.15)	80792 (61.74)	11484 (68.09)
Yes	15301 (45.76)	6080 (28.85)	50059 (38.26)	5383 (31.91)
Diabetes, *n* (%)				
No	28064 (83.94)	19312 (91.65)	115471 (88.25)	15689 (93.02)
Yes	5370 (16.06)	1760 (8.35)	15380 (11.75)	1178 (6.98)
Heart disease, *n* (%)				
No	27861 (83.33)	18126 (86.02)	113910 (87.05)	14498 (85.95)
Yes	5573 (16.67)	2946 (13.98)	16941 (12.95)	2369 (14.05)
Stroke, *n* (%)				
No	31513 (94.25)	20448 (97.04)	125393 (95.83)	16360 (96.99)
Yes	1921 (5.75)	624 (2.96)	5458 (4.17)	507 (3.01)
Any IADL impairment, *n* (%)				
No	29215 (87.38)	17123 (81.26)	120090 (91.78)	15116 (89.62)
Yes	4219 (12.62)	3949 (18.74)	10761 (8.22)	1751 (10.38)
IADL ≥2 impairments, *n* (%)				
No	31657 (94.69)	19307 (91.62)	125777 (96.12)	16177 (95.91)
Yes	1777 (5.31)	1765 (8.38)	5074 (3.88)	690 (4.09)
Cognition (baseline), mean (SD)				
IMRC	5.52 (1.71)	3.93 (1.73)	5.09 (1.85)	5.71 (1.80)
DLRC	4.43 (2.05)	3.52 (2.31)	3.63 (2.13)	4.35 (2.13)
TR20	9.94 (3.53)	7.45 (3.64)	8.72 (3.70)	10.05 (3.65)

IADL, instrumental activities of daily living; IMRC, immediate recall score; DLRC, delayed recall score; TR20, total recall score calculated as IMRC + DLRC and reported descriptively; SD, standard deviation. Values are presented as mean (SD) for continuous variables and *n* (%) for categorical variables.

### Functional ability

Functional status was assessed using self-reported difficulty in instrumental activities of daily living. Across cohorts, the harmonized IADL domains included five overlapping instrumental activities: telephone use, medication management, money management, shopping, and meal preparation. Each item was recoded as impaired versus unimpaired, and an IADL impairment count was created by summing impaired domains when at least three of the five items were observed. Any IADL impairment was defined as difficulty in one or more IADL domains. This harmonized definition was applied consistently across all cohorts (Reppermund et al., 2013). The original cohort questionnaires used cohort-specific wording and response options to assess difficulty in these domains. To maximize comparability, responses were dichotomized into difficulty versus no difficulty before calculating the impairment count. Because the source surveys did not harmonize severity, assistance, non-performance, or reasons for difficulty in the same way across cohorts, the primary exposure should be interpreted as a pragmatic binary indicator of reported difficulty rather than a fully graded measure of functional disability. Additional details on variable harmonization are summarized in Supplementary Table S2.

### Covariates

All primary models were adjusted for baseline age, sex, education, current smoking, hypertension, diabetes, heart disease, and stroke. These variables were selected *a priori* as potential confounders of the association between functional impairment and cognitive change. Age was modeled in years, sex was self-reported, and education was harmonized into three categories: below high school, high school, and above high school. Below high school served as the reference category in adjusted models. Smoking and chronic disease variables were modeled as binary indicators.

### Statistical analysis

We used longitudinal cohort-specific analyses followed by random-effects meta-analysis to summarize replicated estimates across cohorts. First, we examined whether baseline IADL impairment was associated with subsequent cognitive slope. In each cohort, we fit linear mixed-effects models with repeated CogZ as the outcome, including follow-up time, baseline IADL impairment, and their interaction (Laird and Ware, 1982). The interaction between baseline IADL impairment and time was interpreted as the difference in annualized cognitive slope associated with baseline functional impairment.

Second, we conducted the primary lagged analysis, in which IADL status at the prior wave was related to subsequent annualized cognitive change between consecutive visits. For each valid interval, annualized cognitive change was calculated as the change in CogZ divided by elapsed follow-up time in years. The exposure was any prior-wave IADL impairment versus none. To evaluate IADL difficulty that emerged after an initially unimpaired state, this analysis was restricted to participants without baseline IADL impairment. Thus, newly emerging IADL impairment in the present study denotes reported IADL difficulty at a follow-up wave among participants who were free of IADL impairment at baseline; it does not require confirmation that the difficulty first appeared in the immediately preceding interval.

Third, we evaluated dose–response associations by replacing the binary prior-wave exposure with the number of impaired IADL domains. We estimated associations for 1 impaired domain versus 0, ≥2 impaired domains versus 0, and per additional impaired domain.

All models were estimated separately within each cohort, and cohort-specific effect estimates were pooled using random-effects meta-analysis (DerSimonian and Laird, 1986). Between-cohort heterogeneity was assessed using Cochran’s *Q* statistic and quantified with the *I*^2^ statistic (Higgins et al., 2003). This two-stage coordinated-analysis approach was chosen to preserve cohort-specific model estimation while providing an overall summary of replicated associations across cohorts. Although the analytic variables were harmonized, the cohorts differed in sampling frame, population structure, survey implementation, cognitive-score distributions, and IADL reporting contexts. Estimating models separately within each cohort therefore allowed cohort-specific effect sizes and heterogeneity to be examined directly, reducing the risk that the pooled result would be driven primarily by the largest cohort. Accordingly, pooled estimates were interpreted together with cohort-specific results rather than as stand-alone estimates of a single universal effect.

### Sensitivity analyses

We conducted prespecified sensitivity analyses to assess the robustness of the primary lagged associations. These analyses addressed practice effects in repeated cognitive testing (Duff et al., 2008; Calamia et al., 2012), selective attrition using inverse probability of censoring weighting (IPCW) (Cole and Hernan, 2008; Robins et al., 2000; Weuve et al., 2012; Seaman and White, 2013), exclusion of participants in the lowest 10% of baseline cognition, exclusion of participants with baseline stroke, and combined specifications incorporating these restrictions. We additionally examined whether the dose–response associations remained stable across these alternative specifications.

### Software

All data management and statistical analyses were performed in R (version 4.4.3). Mixed-effects models were fitted using standard longitudinal modeling packages, and random-effects meta-analyses were conducted using the metafor package.

## Results

### Participant characteristics and analytic sample

A total of 202,224 participants from CHARLS, ELSA, HRS, and SHARE met the criteria for inclusion in the analytic sample. Among them, 114,039 individuals contributed 338,308 valid follow-up intervals to the primary lagged analysis. The derivation of the analytic sample is shown in Figure 1, and baseline characteristics of the included participants are summarized in Table 1. Stepwise cohort-specific sample selection is provided in Supplementary Table S1. The cohorts differed meaningfully in baseline composition. SHARE contributed the largest and oldest sample, with a mean age of 64.22 years and 44.32% of participants aged ≥65 years. CHARLS participants were younger on average, with a mean age of 59.64 years, but had the highest proportion with education below high school, the highest prevalence of current smoking, and the highest prevalence of any IADL impairment. HRS participants had the highest prevalence of hypertension, diabetes, and stroke.

### Prior-wave IADL impairment predicts accelerated cognitive decline

**Figure 2:**
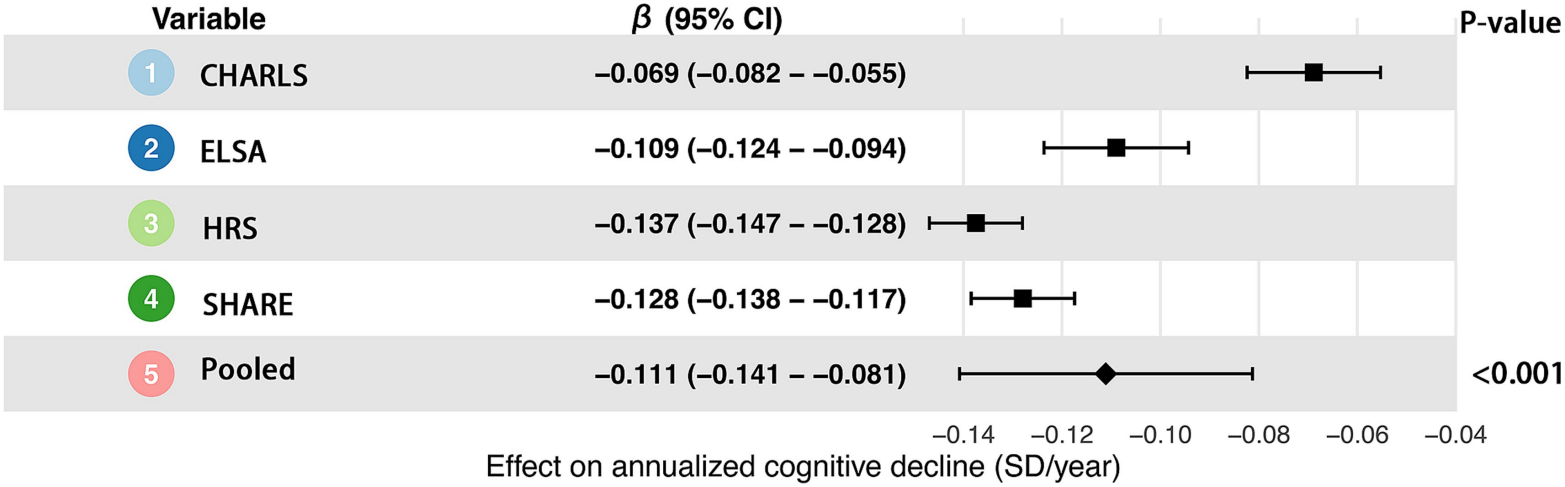
Association of prior-wave IADL impairment with subsequent annualized cognitive decline across four population-based cohorts. Forest plot showing cohort-specific and pooled estimates for the association between any prior-wave IADL impairment and subsequent annualized change in global cognition. Prior-wave IADL impairment was defined as difficulty in one or more instrumental activities of daily living at the preceding assessment. Cohort-specific estimates are shown for CHARLS, ELSA, HRS, and SHARE, and the pooled estimate was obtained using a random-effects meta-analysis. Squares represent cohort-specific effect estimates, horizontal lines indicate 95% confidence intervals, and the pooled estimate is shown separately. Negative values indicate faster subsequent cognitive decline among participants with prior-wave IADL impairment.

In the primary lagged analysis, prior-wave IADL impairment was associated with faster subsequent annualized decline in global cognition. Participants with any IADL impairment at the prior assessment showed significantly steeper decline than those without prior-wave impairment (pooled *β* = −0.111 SD/year, 95% CI −0.141 to −0.081, *p* < 0.001), with substantial between-cohort heterogeneity (*I*^2^ = 96.1%) (Figure 2; Table 2). For clinical interpretation, this slope difference corresponds to an expected additional difference of approximately −0.22 SD over 2 years, −0.44 SD over 4 years, and −0.67 SD over 6 years, assuming the estimated annualized difference remains constant over time.

**Table 2:** Pooled associations of IADL impairment with subsequent cognitive decline across CHARLS, ELSA, HRS, and SHARE.

Analysis	Comparison	Pooled *β* (95% CI)	*p* value	*I*^2^, %
Primary lagged analysis	Any prior-wave IADL impairment vs. none	−0.111 (−0.141 to −0.081)	<0.001	96.1
Dose–response analysis	1 impaired domain vs. 0	−0.095 (−0.118 to −0.072)	<0.001	90.8
	≥2 impaired domains vs. 0	−0.147 (−0.192 to −0.103)	<0.001	95
	Per additional impaired domain	−0.063 (−0.081 to −0.045)	<0.001	96.7
Baseline IADL analysis	Baseline IADL impairment × time	0.005 (−0.002 to 0.011)	0.145	89.4

IADL, instrumental activities of daily living; CI, confidence interval.

This association was directionally consistent across all four cohorts but varied in magnitude. Cohort-specific estimates were −0.075 (95% CI −0.088 to −0.061) in CHARLS, −0.102 (95% CI −0.118 to −0.087) in ELSA, −0.127 (95% CI −0.136 to −0.117) in HRS, and −0.129 (95% CI −0.140 to −0.119) in SHARE. The corresponding numbers of valid follow-up intervals were 30,800, 53,856, 111,449, and 142,203, respectively. Thus, the association was weakest in CHARLS and strongest in HRS and SHARE, contributing to the high *I*^2^ value while preserving a consistent negative direction across cohorts (Table 3). We therefore interpreted the pooled estimate as a summary of replicated cohort-specific associations, not as evidence of a single universal effect size.

**Table 3:** Cohort-specific associations of IADL impairment with subsequent cognitive decline.

Analysis	Comparison	CHARLS *β* (95% CI)	ELSA *β* (95% CI)	HRS *β* (95% CI)	SHARE *β* (95% CI)	Pooled *β* (95% CI)
Primary lagged analysis	Any prior-wave IADL impairment vs. none	−0.075 (−0.088 to −0.061)	−0.102 (−0.118 to −0.087)	−0.127 (−0.136 to −0.117)	−0.129 (−0.140 to −0.119)	−0.111 (−0.141 to −0.081)
Dose–response analysis	1 impaired domain vs. 0	−0.066 (−0.082 to −0.049)	−0.092 (−0.110 to −0.075)	−0.107 (−0.119 to −0.096)	−0.104 (−0.117 to −0.092)	−0.095 (−0.118 to −0.072)
	≥2 impaired domains vs. 0	−0.092 (−0.114 to −0.070)	−0.129 (−0.159 to −0.099)	−0.166 (−0.181 to −0.151)	−0.175 (−0.192 to −0.158)	−0.147 (−0.192 to −0.103)
	Per additional impaired domain	−0.040 (−0.047 to −0.032)	−0.064 (−0.074 to −0.054)	−0.072 (−0.077 to −0.066)	−0.067 (−0.072 to −0.061)	−0.063 (−0.081 to −0.045)
Baseline IADL analysis	Baseline IADL impairment × time	0.001 (—)	−0.003 (—)	0.008 (—)	0.012 (—)	0.005 (−0.002 to 0.011)

IADL, instrumental activities of daily living; CI, confidence interval. Negative values indicate faster subsequent cognitive decline. Cohort-specific estimates were derived from models fitted separately within each cohort; pooled estimates were obtained using random-effects meta-analysis.

### Dose–response association according to IADL impairment burden

A graded association was observed according to the number of impaired IADL domains. Compared with having no impaired IADL domains at the prior assessment, having one impaired domain was associated with faster subsequent cognitive decline (pooled *β* = −0.095 SD/year, 95% CI −0.118 to −0.072, *p* < 0.001), whereas having two or more impaired domains was associated with an even steeper decline (pooled *β* = −0.147 SD/year, 95% CI −0.192 to −0.103, *p* < 0.001). When impairment burden was modeled continuously, each additional impaired IADL domain was associated with faster annualized decline (pooled *β* = −0.063 SD/year, 95% CI −0.081 to −0.045, *p* < 0.001) (Table 2).

The cohort-specific estimates showed the similar gradient. For the comparison of 1 impaired domain versus 0, estimates ranged from −0.066 in CHARLS to −0.107 in HRS. For the comparison of ≥2 impaired domains versus 0, estimates ranged from −0.092 in CHARLS to −0.175 in SHARE. When modeled continuously, each additional impaired domain was associated with faster decline in every cohort, with estimates ranging from −0.040 in CHARLS to −0.072 in HRS. These cohort-specific and pooled estimates are reported in Table 3, and forest plots are shown in Supplementary Figure S2.

### Baseline IADL impairment alone does not predict subsequent cognitive slope

By contrast, baseline IADL impairment alone was not significantly associated with subsequent cognitive slope. In mixed-effects models of repeated CogZ over follow-up, the interaction between baseline IADL impairment and time was not statistically significant in the pooled analysis (pooled *β* = 0.005 SD/year, 95% CI −0.002 to 0.011, *p* = 0.145), despite substantial between-cohort heterogeneity (*I*^2^ = 89.4%) (Table 2).

Cohort-specific estimates were small and inconsistent in direction, with values of 0.001 in CHARLS, −0.003 in ELSA, 0.008 in HRS, and 0.012 in SHARE. These findings suggest that baseline functional status alone was less informative than IADL difficulties that emerged during follow-up among initially unimpaired participants. Corresponding cohort-specific results are shown in Table 3, Supplementary Figure S2.

### Sensitivity analyses

The association between prior-wave IADL impairment and accelerated annualized cognitive decline remained robust across prespecified sensitivity analyses. The pooled estimate remained negative after exclusion of participants in the lowest decile of baseline cognition (*β* = −0.101 SD/year, 95% CI −0.126 to −0.076), after accounting for informative attrition using inverse probability of censoring weighting (*β* = −0.111 SD/year, 95% CI −0.138 to −0.084), and after adjustment for practice effects in repeated cognitive testing (*β* = −0.104 SD/year, 95% CI −0.110 to −0.097). The combined specification incorporating both practice-effect adjustment and attrition weighting also yielded a similar estimate (*β* = −0.106 SD/year, 95% CI −0.112 to −0.100). Under the most stringent specification, which additionally excluded participants in the lowest baseline cognition decile and participants with baseline stroke, the association remained materially unchanged (*β* = −0.101 SD/year, 95% CI −0.107 to −0.094).

The dose–response findings were likewise preserved across alternative specifications. Under the most stringent specification, the comparison of 1 impaired domain versus 0 remained statistically significant (*β* = −0.081 SD/year, 95% CI −0.089 to −0.073), as did the comparison of ≥2 impaired domains versus 0 (*β* = −0.137 SD/year, 95% CI −0.151 to −0.124) and the per-domain association (*β* = −0.055 SD/year, 95% CI −0.060 to −0.050). The full set of sensitivity-analysis results is presented in Supplementary Figure S1, Supplementary Table S4.

## Discussion

### Summary of principal findings

In this harmonized multi-cohort study spanning China, England, the United States, and continental Europe, we identified a temporal pattern linking IADL difficulty emerging during follow-up to subsequent decline in global cognition. Baseline IADL impairment alone was not a reliable predictor of long-term cognitive slope, whereas follow-up IADL difficulty among initially unimpaired participants preceded faster decline and showed a graded dose–response relationship. Although the magnitude of the association varied across cohorts, the direction of effect was consistent. These findings support repeated IADL assessment as a marker of heightened cognitive vulnerability, while emphasizing risk stratification rather than causal prediction of neurodegenerative decline.

These results address an important prevention-oriented need. Dementia prevalence is projected to rise substantially worldwide (GBD 2019 Dementia Forecasting Collaborators, 2022), and major policy and research frameworks increasingly emphasize earlier identification of individuals at elevated risk and the implementation of scalable prevention strategies, particularly in settings where biomarker access remains limited (Livingston et al., 2017, 2020, 2024; Karikari et al., 2020). Our findings indicate that repeated IADL assessment, based on brief and low-cost functional questions, may provide a pragmatic way to identify individuals who warrant closer cognitive follow-up. Because the present study used observational cohort data, IADL change should be viewed as a risk-stratification signal rather than a disease-specific marker.

### Comparison with existing evidence

IADLs were originally conceptualized as complex activities required for independent living and distinct from basic self-care (Lawton and Brody, 1969). Early clinical descriptions of MCI emphasized relative preservation of everyday functioning (Petersen et al., 1999; Winblad et al., 2004), although later diagnostic guidance acknowledged that subtle functional changes may already be present and may be important for differentiating MCI from dementia (Albert et al., 2011). In line with this conceptual evolution, a systematic review found that individuals with MCI show measurable deficits in complex IADLs across multiple studies, particularly in tasks placing high demands on memory, planning, and executive control (Jekel et al., 2015). Prospective evidence further supports the prognostic value of everyday function. Tabert et al. (2002) showed that functional deficits among patients with MCI predicted subsequent Alzheimer’s disease dementia, while population-based studies from Germany and Leipzig found that IADL impairment and MCI were associated with incident dementia and shorter time to dementia onset (Luck et al., 2011, 2012).

Our study extends this literature in several important ways. First, by applying a wave-to-wave lagged design, we moved beyond concurrent associations and showed that IADL impairment at an earlier time point was associated with faster subsequent cognitive decline. This temporal ordering is consistent with prior longitudinal evidence suggesting that restriction in complex activities is detectable in MCI and may emerge years before the clinical diagnosis of dementia (Peres et al., 2006, 2008). However, this temporal ordering cannot fully exclude reverse causality, because subtle cognitive decline may already have been present before IADL difficulty was reported, particularly given approximately 2-year assessment intervals and brief cognitive measures. Second, we provided cross-national replication across four large population-based aging cohorts, showing that the association was directionally consistent across diverse settings even though the magnitude of effect varied (Zhao et al., 2014; Steptoe et al., 2013; Sonnega et al., 2014; Börsch-Supan et al., 2013). Third, the observed dose–response gradient strengthened the interpretation that increasing functional burden reflects increasing cognitive vulnerability, rather than a simple threshold effect.

Our findings are also consistent with prior work indicating that cognitively demanding IADL tasks are particularly sensitive to early cognitive vulnerability. In the Sydney Memory and Ageing Study, impairment in IADLs with high cognitive demand was an early marker of MCI (Reppermund et al., 2013), and Farias et al. (2006) reported that MCI was associated with deficits in everyday functioning, especially for tasks requiring complex cognitive processing. Studies using the Functional Activities Questionnaire have shown that specific functional items can discriminate subtle impairment and predict progression from clinically normal status to MCI (Pfeffer et al., 1982; Marshall et al., 2015). Taken together, the current results reinforce the view that incident difficulty in complex daily activities should not be regarded as a nonspecific accompaniment of aging, but rather as a signal warranting closer cognitive evaluation.

### Biological plausibility and mechanistic interpretation

Contemporary models of Alzheimer’s disease describe a long preclinical and prodromal phase during which neuropathological changes accumulate before overt dementia becomes clinically apparent (Sperling et al., 2011; Jack et al., 2016, 2018; McKhann et al., 2011; Dubois et al., 2014). Within this framework, IADL impairment is biologically plausible as an early manifestation because complex everyday tasks depend on the coordinated functioning of episodic memory, attention, executive control, and processing speed. Difficulty in these tasks may therefore emerge when cognitive systems become sufficiently vulnerable, even before more global disability is recognized. Nevertheless, the cognitive outcome available across all cohorts was based on immediate recall, delayed recall, and a brief mental-status/executive component. The observed association may therefore reflect memory-dominant cognitive decline more strongly than global cognition measured by a comprehensive neuropsychological battery. Viewed with this boundary, repeated IADL assessment may capture behaviorally expressed cognitive vulnerability before more overt clinical impairment becomes apparent.

Biomarker-linked evidence supports the plausibility of this interpretation. In symptomatic Alzheimer’s disease, IADL impairment has been associated with cortical amyloid burden and tau accumulation, particularly in inferior and medial temporal regions (Halawa et al., 2019). In the A4 Study screening sample, “cognitively normal” referred to older adults without a clinical diagnosis of cognitive impairment or dementia at screening; within this group, subtle IADL difficulties were associated with amyloid burden and lower cognitive performance (Marshall et al., 2020). Complementary work suggests that decline in cognitively complex everyday activities accelerates along the Alzheimer’s disease continuum (Dubbelman et al., 2020a). Because our analyses did not incorporate biomarker stratification or adjudicated dementia outcomes, the temporal pattern should not be interpreted as direct evidence that incident IADL impairment reflects Alzheimer’s disease-specific neurodegeneration.

From a translational perspective, these functional signals may complement emerging molecular markers. Emerging blood-based biomarkers, particularly phosphorylated tau assays, illustrate the field’s move toward more scalable biological assessment of Alzheimer’s disease-related pathology (Karikari et al., 2020). However, AD-specific biomarker data were not harmonized and longitudinally available across all four cohorts and waves used in the present analysis. In practice, repeatable IADL screening may therefore serve as a low-cost front-end tool to identify individuals who may benefit from more detailed cognitive assessment and, where available, biomarker evaluation.

### Cross-national heterogeneity and measurement considerations

Although the direction of association was consistent, between-cohort heterogeneity was substantial and should temper interpretation of the pooled estimate. Cohort-specific results indicated that prior-wave IADL impairment was associated with faster subsequent decline in all cohorts, but the estimated magnitude was smaller in CHARLS and larger in HRS and SHARE. This pattern should be interpreted in the context of baseline differences: CHARLS had markedly lower educational attainment and a higher prevalence of baseline IADL impairment, SHARE was older on average and had the largest proportion of participants aged ≥65 years, and HRS had a higher cardiometabolic and stroke burden. Educational level, cultural expectations, survey implementation, and health-system context may modify both the likelihood of reporting IADL difficulty and the extent to which a new IADL difficulty reflects cognitive vulnerability rather than physical limitation, long-standing role allocation, or contextual demand. This is not unexpected in a pooled analysis spanning countries with differences in population age structure, educational attainment, comorbidity burden, health systems, and survey implementation (Zhao et al., 2014; Steptoe et al., 2013; Sonnega et al., 2014; Börsch-Supan et al., 2013). Functional measures are particularly sensitive to social and cultural context, because expectations regarding household roles, financial management, shopping, and telephone use may differ across settings. More broadly, the cross-cultural adaptation of self-report measures is itself a methodologically important process (Beaton et al., 2000).

Evidence from international validation of the Amsterdam IADL Questionnaire suggests that item bias attributable to diversity can be limited in carefully developed instruments, while also highlighting the need for explicit measurement evaluation across settings (Dubbelman et al., 2020b). Our harmonized binary definition of IADL impairment enhanced comparability but necessarily simplified a multidimensional construct. Dichotomization may lose information about severity, assistance, non-performance, and reasons for difficulty. In population cohorts, difficulty in an IADL task may reflect not only cognitive vulnerability but also physical limitations, environmental constraints, gendered or cultural role allocation, lack of opportunity to perform a task, or differences in household responsibility. This limitation is inherent to large epidemiologic studies and likely contributed to residual heterogeneity.

At the same time, the broader literature supports the interpretive relevance of functional change. Beyond brief general-population measures, several dementia-oriented instruments have been developed and validated, including the Functional Activities Questionnaire, the Everyday Cognition scale, and the Amsterdam IADL Questionnaire (Pfeffer et al., 1982; Farias et al., 2008; Sikkes et al., 2013a,b; Koster et al., 2015; Jutten et al., 2017). These instruments underscore both the diagnostic value of functionally focused assessment and the importance of measurement properties when comparing results across studies and populations.

### Why baseline IADL impairment differed from time-varying (incident) IADL impairment

One of the most informative findings in this study was the contrast between the null pooled result for baseline IADL impairment and the robust associations observed for prior-wave impairment among participants who were unimpaired at baseline. This distinction is clinically plausible and can be understood as a contrast between static impairment and functional decline. Baseline IADL impairment in population cohorts is likely to be etiologically heterogeneous and may reflect chronic physical limitations, contextual factors, stable long-standing impairments, or social role patterns that are not tightly coupled to subsequent cognitive decline. By contrast, IADL difficulty emerging after an initially unimpaired state captures change in everyday function. Functional change may be more closely aligned with a recent shift in cognitive reserve, health status, or underlying disease processes than a single static baseline measure.

This interpretation is consistent with evidence showing that everyday functioning changes over time in individuals with early cognitive vulnerability (Wadley et al., 2007) and that decline in cognitively complex daily activities accelerates along the Alzheimer’s disease continuum (Dubbelman et al., 2020a). Conceptually, a static baseline measure combines prevalent, long-standing, and potentially non-cognitive sources of impairment, whereas a repeated measure can detect a transition from intact to impaired everyday function. In this sense, time-varying IADL impairment may be more informative than a single baseline assessment because it captures change, and change itself may be more relevant than static status when identifying individuals entering a period of accelerated decline.

### Clinical and public-health implications

These findings support the use of repeatable IADL assessment as a scalable approach to monitoring cognitive aging and identifying older adults who may benefit from more detailed cognitive evaluation. Given the projected growth in dementia burden (GBD 2019 Dementia Forecasting Collaborators, 2022) and the emphasis on prevention and early identification in major commissions and policy recommendations (Livingston et al., 2017, 2020, 2024), a pragmatic approach is to treat incident IADL impairment as a trigger for stepped follow-up in community and primary care. Such follow-up could include brief cognitive testing, review of reversible contributors such as depressive symptoms or medication effects, vascular risk assessment, and closer longitudinal monitoring. When available and clinically appropriate, referral for specialist assessment or biomarker evaluation may be considered. This stepwise framing preserves the translational value of IADL screening while avoiding overstatement of IADL impairment as a disease-specific marker.

This implication is especially relevant in settings where biomarker access remains limited or uneven (Karikari et al., 2020). While functional questions do not replace biomarker-based characterization, they may provide an accessible front-line screening tool that improves triage efficiency and broadens the reach of early detection strategies. The target population for this inference is adults who are initially free of IADL impairment; the findings should not be generalized directly to individuals with chronic, long-standing, or baseline functional limitations.

### Strengths and limitations

This study has several strengths. It included a large combined sample drawn from four well-characterized population-based aging cohorts, used harmonized definitions and a coordinated analytic strategy, and applied a time-lagged design that strengthened temporal interpretation. The use of prespecified sensitivity analyses addressing practice effects and selective attrition further supports the robustness of the findings (Duff et al., 2008; Calamia et al., 2012; Cole and Hernan, 2008; Robins et al., 2000; Weuve et al., 2012; Seaman and White, 2013).

Several limitations should also be acknowledged. First, IADL was measured by self-report, which may be influenced by reporting bias, physical limitations, gendered or cultural role allocation, environmental constraints, and cultural context (Beaton et al., 2000; Dubbelman et al., 2020b; Zhao et al., 2014; Higgins et al., 2003). Second, the available IADL items were only partially equivalent across cohorts, and harmonization necessarily required simplification. The binary impairment definition did not preserve comparable information on severity, assistance, non-performance, or reasons for difficulty. Third, the cognitive outcome was harmonized from immediate recall, delayed recall, and a brief mental-status/executive component; it may therefore overweight memory and may not capture executive decline as comprehensively as a full neuropsychological battery. Fourth, residual confounding and reverse causality remain possible because subtle cognitive decline may have preceded reported IADL difficulty. Fifth, AD-specific biomarker data and adjudicated dementia outcomes were not harmonized and longitudinally available across all four cohorts and waves, preventing direct evaluation of whether incident IADL impairment predicted biomarker change or clinical conversion. Finally, the high I^2^ values indicate substantial between-cohort inconsistency, and the random-effects variance was necessarily estimated imprecisely with four cohorts; pooled estimates should therefore be interpreted alongside the cohort-specific results rather than in isolation (Higgins et al., 2003).

### Future research directions

Future work should integrate repeated IADL assessment with adjudicated clinical outcomes and, where feasible, biomarker measures to clarify how incident IADL impairment maps onto biological staging frameworks and clinically meaningful transition points (Sperling et al., 2011; Jack et al., 2016, 2018; McKhann et al., 2011; Dubois et al., 2014; Karikari et al., 2020; Halawa et al., 2019; Marshall et al., 2020; Huang et al., 2023; Dubbelman et al., 2020a). Biomarker-enriched subsets of aging cohorts could test whether newly emerging IADL difficulty predicts longitudinal change in amyloid, tau, neurodegeneration, or blood-based biomarker profiles. Cross-cultural psychometric evaluation and invariance testing would further strengthen comparability across regions (Beaton et al., 2000; Dubbelman et al., 2020b). Intervention studies are also needed to determine whether IADL-triggered stepped-care pathways improve cognitive, functional, and care-planning outcomes.

## Conclusion

Across four large population-based cohorts from China, England, the United States, and continental Europe, prior-wave IADL impairment among initially unimpaired adults was associated with accelerated subsequent decline in harmonized cognition. This association was directionally consistent across cohorts, strengthened with increasing IADL impairment burden, and remained robust across multiple sensitivity analyses, although effect magnitudes were heterogeneous. By contrast, baseline IADL impairment alone was not a reliable predictor of subsequent cognitive slope. These findings support newly emerging IADL difficulty as a scalable marker for risk stratification and timely cognitive evaluation, while underscoring the need for cautious interpretation because self-reported IADL difficulty is not disease specific.

## Data Availability

The datasets presented in this study can be found in online repositories. The names of the repository/repositories and accession number(s) can be found in the article/Supplementary material.
