# Supplementary material for: Newly emerging IADL impairment predicts subsequent cognitive decline across four population-based aging cohorts
Source: Front Aging Neurosci. 2026 Jul 22;18:1829741. doi: 10.3389/fnagi.2026.1829741 (PMC13437713; doi:10.3389/fnagi.2026.1829741)
Supplement: Supplementary file 1 [file Table_1.DOCX]

**Supplementary Materials**

**Title: Incident Instrumental Activities of Daily Living Impairment and Subsequent Cognitive Decline: Evidence from CHARLS, ELSA, HRS, and SHARE**

Xiaofan Xu^1†^, Jie Hao^2†^, Yan Fan^1^, Yiming Huang^3^, Zhenkun Huang^2#^, Huiyuan Zhang^1#^

1. Department of Neurology, Liaocheng People’s Hospital, Liaocheng, Shandong, China

2. Department of Neurology, Liaocheng Veterans Hospital of Shandong Province, Liaocheng, Shandong, China

3. Department of Neurology, Xuanwu Hospital, Capital Medical University, Beijing 100053, China

This Supplementary Material includes:

**Supplementary Table S1.** Stepwise construction of the analytic sample in CHARLS, ELSA, HRS, and SHARE

**Supplementary Table S2.** Harmonization of cognition and instrumental activities of daily living measures across CHARLS, ELSA, HRS, and SHARE

**Supplementary Table S3.** Cohort-specific associations of IADL impairment with subsequent cognitive decline

**Supplementary Table S4.** Sensitivity analyses for the association between prior-wave IADL impairment and subsequent cognitive decline

**Supplementary Figure S1.** Sensitivity analyses for the associations of prior-wave IADL impairment with subsequent cognitive decline across four ageing cohorts

**Supplementary Figure S2.** Cohort-specific and pooled associations for the primary and dose-response analyses across CHARLS, ELSA, HRS, and SHARE

| Supplementary Table S1. Stepwise construction of the analytic sample in CHARLS, ELSA, HRS, and SHARE | | | | | |
| --- | --- | --- | --- | --- | --- |
| Selection step | CHARLS | ELSA | HRS | SHARE | Total |
| Participants available in the source datasets | 25,873 | 19,802 | 36,529 | 136,843 | 219,047 |
| Age-eligible participants (aged ≥50 years) | 23,687 | 19,256 | 35,808 | 134,317 | 213,068 |
| Excluded because no eligible baseline assessment was available | 2,615 | 2,389 | 2,374 | 3,466 | 10,844 |
| Participants included in the analytic sample | 21,072 | 16,867 | 33,434 | 130,851 | 202,224 |
| Excluded because no post-baseline follow-up visit was available | 5,458 | 3,506 | 2,695 | 50,612 | 62,271 |
| Participants with at least one post-baseline follow-up visit | 15,614 | 13,361 | 30,739 | 80,239 | 139,953 |
| Excluded from the primary lagged analysis because of baseline IADL impairment | 3,949 | 1,751 | 4,219 | 10,761 | 20,680 |
| Participants without baseline IADL impairment | 17,123 | 15,116 | 29,215 | 120,090 | 181,544 |
| Participants contributing at least one valid follow-up interval to the primary lagged analysis | 11,281 | 11,828 | 23,431 | 67,499 | 114,039 |
| Valid follow-up intervals included in the primary lagged analysis | 30,800 | 53,856 | 111,449 | 142,203 | 338,308 |
| Abbreviations: CHARLS, China Health and Retirement Longitudinal Study; ELSA, English Longitudinal Study of Ageing; HRS, Health and Retirement Study; SHARE, Survey of Health, Ageing and Retirement in Europe; IADL, instrumental activities of daily living. | | | | | |
| Note: The analytic sample comprised participants with an eligible baseline assessment, defined as the first wave with sufficient information on IADL status, cognition, covariates, and interview timing. The primary lagged analysis was further restricted to participants without baseline IADL impairment and to valid follow-up intervals meeting prespecified interval criteria. | | | | | |

| Supplementary Table S2. Harmonization of cognition and instrumental activities of daily living measures across CHARLS, ELSA, HRS, and SHARE | | | | | |
| --- | --- | --- | --- | --- | --- |
| Domain | CHARLS | ELSA | HRS | SHARE | Harmonized analytic use |
| Cohort | China Health and Retirement Longitudinal Study | English Longitudinal Study of Ageing | Health and Retirement Study | Survey of Health, Ageing and Retirement in Europe | Four population-based ageing cohorts analyzed separately and pooled |
| Participant age threshold | Analytic code used age ≥50 | Age ≥50 | Age ≥50 | Age ≥50 | Age-eligible analytic sample |
| Cognitive component 1 | Immediate word recall (imrc) | Immediate word recall (imrc) | Immediate word recall (imrc) | Immediate word recall (imrc) | Standardized within cohort |
| Cognitive component 2 | Delayed word recall (dlrc) | Delayed word recall (dlrc) | Delayed word recall (dlrc) | Delayed word recall (dlrc) | Standardized within cohort |
| Cognitive component 3 | Mental status / orientation-executive item set (tr20) | Mental status / orientation-executive item set (tr20) | Mental status / orientation-executive item set (tr20) | Mental status / orientation-executive item set (tr20) | Standardized within cohort |
| Global cognition outcome | CogZ from mean of available z-scores (≥2 components required) | CogZ from mean of available z-scores (≥2 components required) | CogZ from mean of available z-scores (≥2 components required) | CogZ from mean of available z-scores (≥2 components required) | Outcome for longitudinal models |
| IADL item 1 | Telephone use | Telephone use | Telephone use | Telephone use | Binary recode; counted if observed |
| IADL item 2 | Medication management | Medication management | Medication management | Medication management | Binary recode; counted if observed |
| IADL item 3 | Money management | Money management | Money management | Money management | Binary recode; counted if observed |
| IADL item 4 | Shopping | Shopping | Shopping | Shopping | Binary recode; counted if observed |
| IADL item 5 | Meal preparation | Meal preparation | Meal preparation | Meal preparation | Binary recode; counted if observed |
| IADL summary measure | Count of impaired IADL domains (0–5) | Count of impaired IADL domains (0–5) | Count of impaired IADL domains (0–5) | Count of impaired IADL domains (0–5) | Exposure for dose-response models |
| Any IADL impairment | ≥1 impaired IADL domain | ≥1 impaired IADL domain | ≥1 impaired IADL domain | ≥1 impaired IADL domain | Binary exposure |
| Minimum observed IADL items required | ≥3 of 5 items | ≥3 of 5 items | ≥3 of 5 items | ≥3 of 5 items | Required to define IADL summary status |
| Standardization reference | Cohort-specific baseline distribution | Cohort-specific baseline distribution | Cohort-specific baseline distribution | Cohort-specific baseline distribution | Reference for component z-scores |
| Abbreviations: IADL, instrumental activities of daily living. | | | | | |
| Note: Immediate recall, delayed recall, and mental status/executive function were standardized within cohort using the baseline distribution of the eligible analytic sample. The global cognition score was defined as the mean of available standardized component scores when at least two of the three components were observed. | | | | | |

| Supplementary Table S3. Cohort-specific associations of IADL impairment with subsequent cognitive decline | | | | | | |
| --- | --- | --- | --- | --- | --- | --- |
| Analysis | Comparison | CHARLS β (95% CI) | ELSA β (95% CI) | HRS β (95% CI) | SHARE β (95% CI) | Pooled β (95% CI) |
| Primary lagged analysis | Any prior-wave IADL impairment vs none | -0.075 (-0.088 to -0.061) | -0.102 (-0.118 to -0.087) | -0.127 (-0.136 to -0.117) | -0.129 (-0.140 to -0.119) | -0.111 (-0.141 to -0.081) |
| Dose-response analysis | 1 impaired domain vs 0 | -0.066 (-0.082 to -0.049) | -0.092 (-0.110 to -0.075) | -0.107 (-0.119 to -0.096) | -0.104 (-0.117 to -0.092) | -0.095 (-0.118 to -0.072) |
| ≥2 impaired domains vs 0 | -0.092 (-0.114 to -0.070) | -0.129 (-0.159 to -0.099) | -0.166 (-0.181 to -0.151) | -0.175 (-0.192 to -0.158) | -0.147 (-0.192 to -0.103) |  |
| Per additional impaired domain | -0.040 (-0.047 to -0.032) | -0.064 (-0.074 to -0.054) | -0.072 (-0.077 to -0.066) | -0.067 (-0.072 to -0.061) | -0.063 (-0.081 to -0.045) |  |
| Baseline IADL analysis | Baseline IADL impairment × time | 0.001 (—) | -0.003 (—) | 0.008 (—) | 0.012 (—) | 0.005 (-0.002 to 0.011) |
| Abbreviations: IADL, instrumental activities of daily living; CI, confidence interval. | | | | | | |
| Note: The primary lagged analysis evaluated whether IADL impairment at the prior wave predicted subsequent annualized cognitive decline. Dose-response models examined the number of impaired IADL domains at the prior wave. Cohort-specific confidence intervals for the baseline IADL analysis were not available in the surfaced result files and are therefore shown as unavailable. | | | | | | |

Supplementary Table S4. Sensitivity analyses for the association between prior-wave IADL impairment and subsequent cognitive decline

| A. Any prior-wave IADL impairment vs none | | | |
| --- | --- | --- | --- |
| Sensitivity analysis | Pooled β (95% CI) | P value | I², % |
| Main model | -0.109 (-0.134 to -0.084) | <0.001 | 94.3 |
| Practice-effect adjustment only | -0.104 (-0.110 to -0.097) | <0.001 | 9.8 |
| IPCW only | -0.111 (-0.138 to -0.084) | <0.001 | 95.2 |
| Practice-effect adjustment + IPCW | -0.106 (-0.112 to -0.100) | <0.001 | 2.7 |
| Excluding the lowest 10% of baseline cognition | -0.101 (-0.126 to -0.076) | <0.001 | 93.5 |
| Excluding baseline stroke | -0.109 (-0.133 to -0.086) | <0.001 | 93.2 |
| Excluding the lowest 10% of baseline cognition and baseline stroke | -0.102 (-0.125 to -0.078) | <0.001 | 92.4 |
| Practice-effect adjustment + IPCW + excluding the lowest 10% of baseline cognition | -0.100 (-0.106 to -0.094) | <0.001 | 0 |
| Practice-effect adjustment + IPCW + excluding baseline stroke | -0.106 (-0.112 to -0.100) | <0.001 | 1.6 |
| Practice-effect adjustment + IPCW + excluding the lowest 10% of baseline cognition and baseline stroke | -0.101 (-0.107 to -0.094) | <0.001 | 0 |

| B. Dose-response: 1 impaired domain vs 0 | | | |
| --- | --- | --- | --- |
| Sensitivity analysis | Pooled β (95% CI) | P value | I², % |
| Main model | -0.093 (-0.112 to -0.075) | <0.001 | 85.3 |
| Practice-effect adjustment only | -0.087 (-0.095 to -0.079) | <0.001 | 15 |
| IPCW only | -0.093 (-0.112 to -0.073) | <0.001 | 86.8 |
| Practice-effect adjustment + IPCW | -0.086 (-0.093 to -0.079) | <0.001 | 1.5 |
| Excluding the lowest 10% of baseline cognition | -0.086 (-0.104 to -0.067) | <0.001 | 84 |
| Excluding baseline stroke | -0.094 (-0.111 to -0.077) | <0.001 | 82.4 |
| Excluding the lowest 10% of baseline cognition and baseline stroke | -0.087 (-0.104 to -0.069) | <0.001 | 81 |
| Practice-effect adjustment + IPCW + excluding the lowest 10% of baseline cognition | -0.080 (-0.087 to -0.073) | <0.001 | 0 |
| Practice-effect adjustment + IPCW + excluding baseline stroke | -0.086 (-0.095 to -0.078) | <0.001 | 28.2 |
| Practice-effect adjustment + IPCW + excluding the lowest 10% of baseline cognition and baseline stroke | -0.081 (-0.089 to -0.073) | <0.001 | 5.9 |

| C. Dose-response: ≥2 impaired domains vs 0 | | | |
| --- | --- | --- | --- |
| Sensitivity analysis | Pooled β (95% CI) | P value | I², % |
| Main model | -0.141 (-0.179 to -0.104) | <0.001 | 93 |
| Practice-effect adjustment only | -0.136 (-0.149 to -0.124) | <0.001 | 40.4 |
| IPCW only | -0.145 (-0.185 to -0.105) | <0.001 | 93.7 |
| Practice-effect adjustment + IPCW | -0.139 (-0.153 to -0.124) | <0.001 | 50.5 |
| Excluding the lowest 10% of baseline cognition | -0.134 (-0.173 to -0.095) | <0.001 | 92.1 |
| Excluding baseline stroke | -0.143 (-0.178 to -0.107) | <0.001 | 91.7 |
| Excluding the lowest 10% of baseline cognition and baseline stroke | -0.136 (-0.173 to -0.098) | <0.001 | 91 |
| Practice-effect adjustment + IPCW + excluding the lowest 10% of baseline cognition | -0.136 (-0.151 to -0.121) | <0.001 | 43.4 |
| Practice-effect adjustment + IPCW + excluding baseline stroke | -0.140 (-0.153 to -0.128) | <0.001 | 31.5 |
| Practice-effect adjustment + IPCW + excluding the lowest 10% of baseline cognition and baseline stroke | -0.137 (-0.151 to -0.124) | <0.001 | 32.6 |

| D. Dose-response: per additional impaired domain | | | |
| --- | --- | --- | --- |
| Sensitivity analysis | Pooled β (95% CI) | P value | I², % |
| Main model | -0.061 (-0.075 to -0.047) | <0.001 | 94.4 |
| Practice-effect adjustment only | -0.057 (-0.062 to -0.053) | <0.001 | 41.4 |
| IPCW only | -0.059 (-0.074 to -0.045) | <0.001 | 94.5 |
| Practice-effect adjustment + IPCW | -0.056 (-0.060 to -0.052) | <0.001 | 34 |
| Excluding the lowest 10% of baseline cognition | -0.058 (-0.073 to -0.044) | <0.001 | 94 |
| Excluding baseline stroke | -0.061 (-0.074 to -0.048) | <0.001 | 93.5 |
| Excluding the lowest 10% of baseline cognition and baseline stroke | -0.059 (-0.073 to -0.045) | <0.001 | 93.2 |
| Practice-effect adjustment + IPCW + excluding the lowest 10% of baseline cognition | -0.055 (-0.060 to -0.050) | <0.001 | 38.6 |
| Practice-effect adjustment + IPCW + excluding baseline stroke | -0.056 (-0.060 to -0.052) | <0.001 | 36.1 |
| Practice-effect adjustment + IPCW + excluding the lowest 10% of baseline cognition and baseline stroke | -0.055 (-0.060 to -0.050) | <0.001 | 41.6 |
| Abbreviations: IPCW, inverse probability of censoring weighting; IADL, instrumental activities of daily living; CI, confidence interval. | | | |
| Note: Sensitivity analyses evaluated the robustness of the lagged association to practice-effect adjustment, selective attrition addressed using IPCW, and exclusion of participants with low baseline cognition and/or baseline stroke. | | | |

Supplementary Figure S1. Sensitivity analyses for the associations of prior-wave IADL impairment with subsequent cognitive decline across four ageing cohorts.

Forest plots showing pooled associations from sensitivity analyses across CHARLS, ELSA, HRS, and SHARE. Panels present results for any prior-wave IADL impairment versus none, 1 impaired domain versus 0, ≥2 impaired domains versus 0, and per additional impaired domain. Sensitivity analyses included adjustment for practice effects, inverse probability of censoring weighting (IPCW), exclusion of participants in the lowest 10% of baseline cognition, exclusion of baseline stroke, and combined models incorporating these restrictions. Across all scenarios, the associations remained directionally consistent, indicating that prior-wave IADL impairment was robustly associated with faster subsequent cognitive decline.

Supplementary Figure S2. Cohort-specific and pooled associations for the primary and dose-response analyses across CHARLS, ELSA, HRS, and SHARE.

Forest plots showing cohort-specific and pooled estimates for the main analyses. Panels present the association of baseline IADL impairment with cognitive change over time, the association of any prior-wave IADL impairment with subsequent cognitive decline, and dose-response analyses comparing 1 impaired domain versus 0, ≥2 impaired domains versus 0, and per additional impaired domain. Baseline IADL impairment alone was not significantly associated with subsequent cognitive decline, whereas prior-wave IADL impairment was associated with faster subsequent decline. The dose-response analyses further showed progressively stronger associations with increasing IADL impairment burden. Squares represent cohort-specific estimates and horizontal lines indicate 95% confidence intervals; diamonds indicate pooled random-effects estimates.
